# Exploring team dynamics during the development of a multi-institutional cross-disciplinary translational team: Implications for potential best practices

**DOI:** 10.1017/cts.2023.640

**Published:** 2023-10-02

**Authors:** Joseph A. Kotarba, Stephen Molldrem, Elise Smith, Heidi Spratt, Suresh K. Bhavnani, Jeffrey S. Farroni, Kevin Wooten

**Affiliations:** 1 Institute for Translational Sciences, University of Texas Medical Branch, Galveston, TX, USA; 2 Department of Sociology, Texas State University, San Marcos, TX, USA; 3 Institute for Bioethics and Health Humanities, University of Texas Medical Branch, Galveston, TX, USA; 4 Department of Biostatistics and Data Science, University of Texas Medical Branch, Galveston, TX, USA; 5 University of Houston Clear Lake, Houston, TX, USA

**Keywords:** Evaluation, multi-institutional, long COVID, team science, translational science, leadership, communication

## Abstract

**Introduction:**

A recent literature review revealed no studies that explored teams that used an explicit theoretical framework for multiteam systems in academic settings, such as the increasingly important multi-institutional cross-disciplinary translational team (MCTT) form. We conducted an exploratory 30-interview grounded theory study over two rounds to analyze participants’ experiences from three universities who assembled an MCTT in order to pursue a complex grant proposal related to research on post-acute sequelae of COVID-19, also called “long COVID.” This article considers activities beginning with preliminary discussions among principal investigators through grant writing and submission, and completion of reviews by the National Center for Advancing Translational Sciences, which resulted in the proposal not being scored.

**Methods:**

There were two stages to this interview study with MCTT members: pre-submission, and post-decision. Round one focused on the process of developing structures to collaborate on proposal writing and assembly, whereas round two focused on evaluation of the complete process. A total of 15 participants agreed to be interviewed in each round.

**Findings:**

The first round of interviews was conducted prior to submission and explored issues during proposal writing, including (1) importance of the topic; (2) meaning and perception of “team” within the MCTT context; and (3) leadership at different levels of the team. The second round explored best practices-related issues including (1) leadership and design; (2) specific proposal assembly tasks; (3) communication; and (4) critical events.

**Conclusion:**

We conclude with suggestions for developing best practices for assembling MCTTs involving multi-institutional teams.

## Introduction

Biomedical research centers increasingly sustain successful multidisciplinary translational teams (MTTs) and multi-institutional cross-disciplinary translational teams (MCTTs) through alignment of continued funding, funding opportunities, recognition of scientific accomplishments, and development and adaptation of team members and team science careers [1,2]. This article reports results from a qualitative 30-interview study with participants from three universities who assembled an MCTT to pursue a complex National Institutes of Health (NIH) grant proposal to study post-acute sequelae of COVID-19 (PASC), also called “long COVID.” The proposal was submitted to the National Center for Advancing Translational Sciences (NCATS) and ultimately not funded.

Our study of team members’ experiences proceeded in two rounds of 15 interviews each; round one was conducted prior to submission of the grant and round two took place after the first review decision. We consider activities beginning with preliminary discussions among principal investigators (PIs) through grant writing, submission, and reactions to reviews. We describe topics related to team dynamics that arose during the composition of the proposal and then after the grant was unscored. We conclude with suggestions for potential best practices in forming future MCTTs.

## Background

Translational research is increasingly conducted by large multidisciplinary teams and multi-university collaborations [3,4]. Research and intellectual property developed by multidisciplinary teams have greater impact and peer recognition than research outputs from siloed investigators [5]. As a result, funding agencies are placing increasing emphasis on team science approaches. A notable example is the Clinical and Translational Sciences Award (CTSA) program of NCATS within the NIH, which is intended to accelerate translational research.

Calhoun and colleagues have proposed an implementation of MTTs using the CTSA infrastructure [1,2,6]. The MTT is a hybrid structure that blends goals of academic researchers with product-driven business teams. Here we examine models, methods, and a case illustration of assessing and evaluating the emerging MCTT model, which emphasizes multi-PI proposals across several institutions.

Considerable progress has been made over the last 20 years in researching the development and effectiveness of translational research teams [7,8]. However, a recent literature review revealed no published studies using the theoretical framework of multiteam systems in academic settings [9]. Multiteam systems are defined by several key characteristics: (1) two or more teams interfacing directly or interdependently; (2) working towards collective goals; (3) acting in response to changing contextual situations and environmental contingencies; (4) team boundaries that are flexible and shifting; (5) independent teams pursuing different proximal goals, but collectively pursuing one distinct goal; and (6) teams that exhibit similar inputs, processes, and outcomes interdependently [10].

Although some evidence suggests that MTTs may create more innovative results [5], other studies suggest that interdisciplinary translational teams are prone to underproduction [11,12]. It thus remains unclear if MTTs or MCTTs are beneficial to research production [1,13]. One effort [14] to understand multiteam systems related to scientific innovation suggests potential difficulties in the combination of team dynamics and processes that operate at different levels of analysis. An example might involve a cohesive team process that increases team productivity, but limits information sharing between teams [13,15]. A multiteam systems approach provides a useful framework to define, study, and operationalize the particularities of the MCTT form.

This qualitative study of a PASC/long COVID MCTT consisted of two rounds of semi-structured interviews with scientists and staff. The MCTT was formed to submit a proposal involving three universities with CTSA grants. These universities are located in different parts of the country and serve distinct populations. One is in a mixed rural/suburban area in the southwest serving a disproportionately Latinx population; another is in rural Appalachia serving a predominantly rural white population; the third is in an urban Rust Belt area serving a predominantly Black population. Leadership at these universities saw synergies between investigators’ interests and their patient populations. So, they formed an MCTT with a Co-PI from each institution to submit a grant in response to an RFP from NCATS to conduct translational PASC/long COVID research. The proposal involved investigators from across the translational pathway, from clinical research and bioinformatics to community engagement.

The goal of the grant proposal was to investigate individuals with Central Nervous System (CNS) symptoms and markers of PASC/long COVID. The first aim was to obtain clinical, behavioral, imaging, bioinformatics, and genomic data from (1) healthy individuals never having had COVID, (2) individuals that have had COVID but no CNS symptoms, and (3) individuals with PASC/long COVID and CNS symptoms. Based on this information, diagnostic criteria would be created for each of these three groups. The second aim was to use national COVID data to create PASC/long COVID criteria using a machine learning approach known as bipartite network analysis. The patients from the first aim would then be classified into groups using results from the bipartite network analysis to better understand the clinical significance of CNS symptoms in relation to PASC/long COVID [14]. The third aim was to utilize a mixed-methods community engagement approach to better understand Social Determinants of Health and social stigma associated with PASC/long COVID to better devise outreach, recruitment, and treatment paradigms.

The leadership at the Institute for Translational Sciences at the University of Texas Medical Branch at Galveston (UTMB) engaged their Team Science Core – of which the authors of this paper are members – to examine the structure and process of this MCTT’s proposal development and submission. Two members of the UTMB study team (Bhavnani and Spratt) were members of the MCTT being studied and were therefore not respondents, but contributed to the reflexive and iterative process of data analysis. The goals were to describe the MCTT’s development and to inform the design of future MCTTs [2].

## Methodology

There were two stages to this interview study with MCTT members: pre-submission, and post-decision. Some respondents participated in both rounds whereas others only in one. Round one focused on the process of developing structures to collaborate on proposal writing and assembly. Invitations were sent to the total population of 38 scientists and staff at all three universities. Fifteen individuals (40% of invitees) agreed to participate in the first round, and semi-structured interviews were conducted from January to March 2021 with the three Co-PIs, six Co-Is, and six staff members. At that time, the team was developing a general idea of the study, considering available and requested resources, and other formative matters.

The proposal was submitted to NIH in July 2021. The second round of interviews was conducted after the proposal was not scored or funded. The purpose of the second round was to understand team members’ perspectives regarding the process, outcome, and team dynamics within this MCTT. Of the original population of 38, only 36 were available for the follow-up interview. Fifteen participants agreed to interviews during the second round, and these were conducted from June 10 to August 27, 2022, consisting of three co-PIs, six Co-Is, and six staff. Fourteen were repeat interviews. The interviews lasted an average of thirty-four minutes. Both rounds of interviews were analyzed through the inductive logic of grounded theory within a constant comparative approach [16] (see Appendices A and B).

We received approval from the UTMB IRB and abided by all relevant regulations (IRB #21-0162). Given the small number of interviews within a tight-knit community of practice, we paid special attention to preserving confidentiality [17]. For example, we only report quotations that are short and non-identifying, while still capturing the overall challenges that the MCTT faced.

## Results

### The First Stage Study: The MCTT in Formation

The initial 15 interviews were conducted with three Co-PIs, six Co-Is, and six staff members while the MCTT prepared the grant. The interviews were conducted and recorded on Zoom, lasting an average of 38 minutes. Respondents were asked about initial steps taken to assemble the proposal, their expectations for its completion, and – hopefully – implementation. We identified three themes from the first round: (1) importance of the research topic; (2) meaning and perception of “team” within the MCTT context; and (3) the importance of leadership at different levels of the team.

### Importance of the Topic

All respondents felt that the proposal topic was timely, for four general reasons. The COVID-19 pandemic was experienced across the entire US population, and more intensely by marginalized groups. Funding opportunities were increasing as government agencies sought to establish research programs, including in terms of long-term effects and care (i.e., PASC/long COVID). The topic is a “fashionable cause,” as several respondents described it. Getting involved with COVID research marked respondents as being – in the words of several different participants – “up-to-date,” “aware of contemporary clinical needs,” aware of “opportunities for institutional growth,” and “commit[ed] to our constituencies.”

A common perception among scientists was that the pandemic provided an ideal context for exercising the principles of translational science with potential benefits for population health [18]. For example, the high-level involvement and value of what are sometimes seen as merely supportive or ancillary elements of biomedical research – such as community engagement, ethics, and staff – were never questioned. There was some legitimate concern voiced, however, that rapid changes in sub-types of SARS-CoV-2 could make current and ongoing research “obsolete.” This perception was especially common among scientists and again reflected the opinion that responses to calls for proposals should be expedited so as to allow existing studies to proceed while also launching new ones.

### Meaning and Perception of “Team” Within the MCTT Context

One enduring area of interest in Team Science is how translational scientists understand the “team” concept [2,19]. All participants explained that the meaning and perception of “team” and its value were affected by the PASC MCTT but for different reasons. These included the effects of trying to coordinate three universities in different locations, the status positions of the universities in higher education, and shortcomings in staffing, among other factors. We observed differences in these respects among scientists and staff. The definitions of “team” used by participants were varied and fluid, revealing that understandings of this key term were more multifarious than most participants’ individual conceptions of the term could accommodate.

However, there were some consistencies across definitions. The scientists felt that – in principle – the design, activities, and staffing of a team ought to be determined by the criteria present in the funding call. As one scientist noted, “We can't invoke personalities much anymore [in assembling teams]. If NCATS says we need a high level of patient involvement, we need to dedicate resources in that direction and find the personnel we need.” In sum, the structure and functioning of translational teams ought to be determined in relation to NCATS’s priorities and institutions’ capacities to execute projects, teammates’ availability, and comfort with collaboration. This approach contrasts with the selection of teammates by personality or likeability.

Staff discovered that the efficiency of the team should be determined in terms of the completion of assigned tasks. The primary example here is “how actual everyday tasks in which staff engage in helping with the proposal do not change as much as with whom they do it,” as one respondent put it. The division of labor in the proposal still required staff at all three universities to do similar tasks and to work together, but they conducted their respective activities somewhat differently. For example, community engagement was handled differently at the three universities, and this problem of misalignment resulted in difficulty in arriving at a shared set of research aims.

The everyday, routine tasks assigned to staff did not change when compared to previous non-MCTT submissions. What did change was who they had to collaborate with across the institutions. Contrasting strengths across the three universities resulted in uneven contributions to the proposal that affected team dynamics, although not uniformly negatively or positively. While respondents acknowledged that the language of “teams” has proliferated across the health sciences, they noted the particular form of the MCTT and the challenges and opportunities that this new style of inter-institutional formation poses for translational science.

### The Importance of Leadership at Different Levels

The Co-PIs worked well together, although this did not translate to well-defined aims. In interviews, junior faculty members and staff assigned to task-oriented teams such as community engagement voiced concern over the difficulty coordinating their activities during proposal assembly. Many participants attributed the failure of the MCTT to arrive at well-defined aims to department-level leadership whose skills in implementing inter-organizational tasks varied. The general expectation was that the learning curve for mastering cross-institutional team dynamics would be short. As a nurse-researcher put it, “I’m not sure how we can improve [our leadership skills]…given the tight deadlines.” This participant and others emphasized that leadership was needed not only from the Co-PIs but throughout the team across all levels. However, the tight submission deadline allowed little time for team formation or “teaming.”

### The Second Stage Study: Post-Decision Reflections

The second round of interviews allowed us to formalize themes from the first stage while also clarifying what participants perceived went well and what went poorly during proposal writing. The topics differed from those in the first round for at least two reasons. First, the interviewer posed different, largely retrospective questions. Second, respondents’ perceptions of the project changed following the unfavorable decision. The original funding call made it clear that researchers could not resubmit unfunded proposals, in contrast to many NIH funding calls that permit resubmission after an unfavorable review. Topics discussed in the second round included (1) leadership and design; (2) specific proposal assembly tasks; (3) communication; and (4) critical events. We explore how each of these issues was discussed by participants.

### Leadership and Design

All respondents spoke positively about the tripartite team structure and leadership. Staff and scientists noted that the Co-PIs were able to get along and build a vision for the project without ego or tensions, although this did not translate to cohesive aims. According to respondents, the leaders were experts in their disciplines and made fairly good decisions in assembling team members. The leaders were aware of the scientific and clinical value of an interdisciplinary translational PASC/long COVID study. In the words of one staff participant, “this is exactly where we should be directing medicine: this is where funding matches need.”

Many MCTT members critiqued the overall proposal design, which resonates with responses from the first round that emphasized difficulty in arriving at shared aims. There was a delay in the MCTT formation and creation of its aims. (The call for proposals was distributed on March 24, 2020; the assembly of the teams began in February, 2021; and the proposal was submitted on July 9, 2021.) This required the MCTT leadership to quickly assemble topical teams, locate resources, generate relationships among scientists and staff, and develop scientific goals. This left little time for the full group to engage in “teaming” activities that could have made the MCTT a more coherent entity. As one participant said, “we thought we all had our lab teams together at our own schools, but we did not have all the labs we needed on the agenda.” Staff worked overtime to prepare documents for internal review and integration. Both scientists and staff agreed that this distress eroded staff morale and proposal quality. The scientists also agreed that they tried to incorporate too many activities. One leader characterized the project as “overly ambitious,” reflecting group consensus.

There was general agreement that horizontal leadership (i.e., across specific research specializations represented at each institution) seemed more important to plan for than high-level leadership (i.e., across Co-PIs). The total list of horizontal or specialty groups included community engagement, genomics, bioinformatics, and imaging. The three Co-PIs generally fulfilled the need for them to communicate often and well among themselves, but relations were more uneven among staff and Co-Is. The aspects of the MCTT that involved horizontal leadership generally involved the execution of specific topics and proposal sections. Horizontal leaders thus had to negotiate different styles of operation and paradigms across institutions and many team members. This especially affected the quality of nonscientific sections of the proposal such as facilities pages and budgets.

Staff noted that relationship work among leadership in MCTTs was more important than in previous translational team configurations; this was partly attributable to the inter-institutional nature of the proposal and MCTT form. Among the staff members who were unsure of their forthcoming role in the proposal assembly, there was a hope and expectation that team leadership would be *charismatic,* an expectation resulting from the innovative nature of the proposal. This affective dimension of leadership was seen as necessary to successfully lead members through unknown territory. Simply being an accomplished scientist was not a sufficient credential for “trail blazing,” as one staff respondent put it. Staff also noted that, in a three-institution partnership with co-equal leadership, the “team” concept too easily evolved into a bureaucratic construct, resulting in less negotiation and more direction. As the project moved along, selling the idea was less critical than working out specific tasks, time schedules, and other logistical matters. Respondents generally felt that these observations would be valuable in planning for subsequent inter-organizational projects, including future MCTTs.

### Specific Proposal Assembly Tasks

According to respondents, the “Writing Team” turned out to be a critical staff-centered team. It consisted of the people who attended the meetings, talked through the ideas, gathered information, and then assembled drafts for review. Writing Team members worked well together and communicated openly across the three universities. The strong working relationships that staff built were largely because they all have experience doing this work in different contexts and understand the expectations of scientific team leaders.

Perhaps the most difficult aspect of the staff-centered team’s work was understanding and integrating the various scientific and lay “languages” used by each institution’s team members. Two members of the writing staff suggested that frequent and specific training in technical writing would have helped them. Some staff also felt they were not getting the morale boosts from the local leadership they thought they needed and deserved.

There was concern among staff that there was not a specific “prime point person” at each school that all personnel could contact for information and direction. This caused numerous issues ranging from questions from participants such as “Whom do I contact regarding IRB?” to “Are we going to do MRIs?” per two respondents. As expected, the scientists were not always available, owing to busy schedules and other factors.

Overall, the medical imaging team was considered an advantage of the proposal. This group was composed of scientists and staff. One scientist noted that the imaging at the three universities “is as good if not better than imaging at the Mayos and the Dukes.”

### Communication

The scientists did not see cross-institutional communications as a major problem. Their sharing of paradigms produced a common language that facilitated conversations and resolutions. For example, communicating with a neurologist at another university is no more difficult than communicating with a neurologist at one’s own university, owing to shared paradigms.

However, the social and behavioral scientists involved with community engagement did not necessarily share paradigms. This was because the community engagement Co-Is came from different disciplinary backgrounds. Therefore, they had difficulty generating productive communication. Some of this was because, although they appear to share research problems and an understanding of the field of community engagement, they worked in somewhat different cultural worlds owing to their regional locations and institutions’ clientele. The everyday life issues facing African Americans in an urban Rust Belt city, Latinx folks in a mixed urban-suburban region in the southwest, and rural Whites in rural Appalachia can vary in intensity, probable solutions, and cultural interpretations of reality. Therefore, the three institutions’ different patient populations were simultaneously a key strength of the proposal and a source of challenges for the MCTT to effectively form. These factors led to communication breakdowns and disconnectedness among the community engagement team members, weakening a critical aspect of the proposal.

Respondents reported little feedback or debriefing of staff by team leadership after the proposal was not funded, as was promised; there was only one brief meeting at each university. PIs are generally geared and conditioned to move from one proposal to the next, while continuously working on existing grants and projects. However, for staff and support personnel, working on a complex, inter-institutional proposal may be their main scheduled task for a relatively lengthy period. Because of the complex, time-constrained, and multi-institutional nature of the proposed research, this MCTT proposal was also seen as an unusually unique and intense experience for staff. We observed more personal investment in this project among staff than among leaders, and this might be a phenomenon to further explore in studies of MCTT formation.

Despite communication issues during proposal assembly, there were overall good feelings among community engagement staff looking ahead toward potential future MCTTs. These feelings were more a result of the collaboration involving shared hard work and dedication than the end product. For example, a senior community engagement leader noted that they were successful in instructing the biomedical scientists that community engagement “is best perceived as an *expertise* and not a *technique*.” To paraphrase this respondent’s view, *expertise* refers to the training, experience, solid theoretical foundation, and especially good judgment that prepares and allows someone to accomplish a specific, complex task. A *technique* is a practical approach to solving a problem that does not require sophisticated decision-making or judgment. The respondent clearly objected to the related perspective that occupations like social workers are less important than medicine.

Communication across the full MCTT turned out to be even more troubled than reported communication challenges among community engagement teams. A common complaint by both staff and scientists was that genomics was generally given top priority in agenda placement and time allotted in meetings. Much of the science was difficult for non-biomedical scientists to comprehend, even following attempts to explain it. This discomfort was exacerbated by having community engagement teams present materials last and with limited time to make their “pitch” in full MCTT meetings.

The leaders all agreed that working together via Zoom was an aggregate plus for them. The contingency, of course, was the COVID-19 pandemic, which made in-person meetings impossible. However, as institutions returned to face-to-face meetings, they realized that meeting via Zoom misses the very important side conversations over coffee, before the meetings, and after the meetings where much scientific discussion and intangible “teaming” work takes place. If future MCTTs wish to integrate an in-person or hybrid model, they will need to budget time and resources accordingly to ensure that an adequate balance of in-person and remote meeting strategies is used to maximum effectiveness.

### Critical Events

In understanding how a group experience like the PASC/long COVID MCTT unfolds, it is valuable to pinpoint events that move the activity in different directions, redefine the activity, support its success, or set up its failure. A sociological concept to address these issues is the *critical event* – described by Garcia-Montoya and Mahoney as “a contingent event that is causally important for an outcome of a particular case” [20]. Participants highlighted several critical events.

Loss and turnover among senior staff were disruptive. These positions were heavily involved in the integration of proposal sections and converting this material into a common language. Negative staff issues, however, were the result of extra-proposal issues beyond the control of the PASC MCTT teams. Therefore, there is very little indication of negative impact from routine and predictable intra-organizational conflict. Loss of one senior scientist was a cause of conflict within the MCTT, but it did not cause the team to collapse; it represented a loss of value in terms of energy, dedication, and substance.

The irony of conducting an experimental project design and team formation (MCTT) that was intended to experiment with theorizing and treating a complicated and novel disease (PASC/long COVID) became increasingly visible during proposal writing. The general sentiment of both biomedical scientists and staff was that future collaborations need to be focused, prepped scientifically and administratively, and aligned with expectations. Working across all levels to enhance shared values among team members is important. As one senior scientist put it, “the MCTT is not the best place to learn how to do science; it is the place to apply one’s scientific skills.”

## Conclusion: Potential Best Practices for Future MCTTs

Overall, respondents largely agreed on general recommendations for future MCTT projects. Potential best practices were generated by the consensus statements made by participants; these are reflected in our recommendations for improved team or team leadership functioning. Interviews and consensual analysis are commonly used qualitative approaches to further understand consensus. [21–24]. This appearance of consensus reflects the positive value of scientists and staff who work closely together on complex proposals. A common impression among participants in both the scientist and staff groups, for example, was that the proposed MCTT was at a disadvantage when competing for valuable grants in a highly competitive field like that of PASC/long COVID. Disadvantages include lack of institutional prestige when compared to the most prestigious research institutions; lack of existing resources to conduct the proposed research; and lack of existing research on PASC/long COVID. A more optimistic view is that the presence of strong competition should encourage thoughtfully designed proposals. A senior scientist in medical imaging neatly summarized this assessment:

I think the group was just too large for producing such a project, and there were too many directions in which that project wanted to go. It should have been probably narrowed down because there were many groups, and this was a grant and clearly three schools, I don't know, for every school, five people. And I think that we wanted to achieve too much with too few patients. And we had neuropsychology, gut imaging, blood biomarkers, I mean just too many things.

This participant’s statement raises fundamental questions about the viability of many large-scale MCTTs. As funders increasingly require more cross-disciplinary and cross-institutional collaboration, it will be important for emergent teams to think carefully about how they are structured and to plan their work with intentionality, including planning adequate time for “teaming” amongst new collaborators. Future MCTTs must keep in mind that they are not only doing science in team formations but that they are also involved in building new forms of multi-institutional translational scientific teams. Table 1 highlights several key takeaways, which can be supported by both general and team science evidence and literature.

**Table 1.** Multi-institutional cross-disciplinary translational teams: a summary

Issues & concerns	Potential best practices	Supporting literature
Establishing a scientific question upon which to base the team	Foster goal clarity/shared mental model: Negotiating a single focus upon which all PIs agree	[25–27]
Designing the structure and staffing of the team	Calibrate team design and staffing: Include a sufficient number of staff/writers who are the keystone of communication	[28–30]
Project budget	Create realistic and flexible budgets: Keep the proposed budget within reasonable limits	[12,31,32]
Including ancillary sub-projects in the proposal	Reduce task and project complexity: Keep them to a minimum because they will likely not receive priority budget or staff	[33–35]
Trust	Build trust: Fostered through open, authentic communication	[36–38]
Leadership	Foster horizontal leadership: Leadership skills should be nurtured both horizontally (among PIs) and vertically (among team leaders)	[39,40]
Contrasting institutional cultures	Appreciate and integrate institutional cultures: Appreciate the fact that your teammates are different from you and live in institutional cultures different from yours	[41–43]
Critical events	Develop situational awareness: Anticipate critical events and deal with them collectively	[44–47]

In interviews, suggestions clustered around comments either from the scientists or the staff members. Scientists, as a group, suggested that future proposals should be more focused on one general topic, such as the microbiome, as opposed to attempting to integrate many methods. They should not, however, begin the proposal process with a total commitment to preestablished aims. Aims should remain fluid as the various sub-teams discuss and debate aims along the way, before solidifying into final aims.

Staff suggested specific recommendations relevant to their goals and needs. Staff whose work focused on budgetary items would have liked the entire team to keep budget constraints in mind throughout the process. Staff agreed that future budgets should consider the value – and limitations – of shifting at least some communication within teams to teleconferencing platforms. Staff also agreed to the value of having a single, knowledgeable contact person at each institution to consult for logistical information, as mentioned above.

Staff from different institutions strongly suggested that they should maintain and grow the relationships and communications channels emanating from the first multi-institutional proposal. They noted that leadership should pay more attention to conflict in their universities, manage it before it enters MCTT activities; or communicate possible effects of conflict on the larger project. MCTT leaders at all levels should realize that the actual team leader is an important *management* position; for example, critical tasks, like the circulation of drafts, need to be monitored by team leaders. The idea that there can be a “one-size-fits-all” approach to community engagement should be abandoned, because the personnel, leadership, histories, geographic settings, and patient populations are quite different across locations. Agreeing on shared principles and objectives for community engagement, while leaving the specifics regarding execution to university-based teams, is a better approach. These findings support Love *et al*.’s [44] recommendation that interaction among team members requires observation and analysis.

The issue of leadership and communication that frequently emerged during the interviews points to the fact that multiteam research is not simply the aggregation of traditional individual teams. Project leadership may have the same staff members as before the MCTT formation, but leadership needs – and thus staff roles – can be very different because of the new, complex roles each staff member must now occupy. Put differently, a PI must now take staff at other universities into consideration when exercising leadership, directly or indirectly, without deep knowledge of the other institution or teams’ culture. These changes in leadership roles may require additional sophisticated training in leadership.

Finally, this MCTT revealed the importance of translational teams taking time to build trust and cohere as a group as they develop. The MCTT we studied had to operate on a rushed timeline with an unfamiliar group of cross-institutional collaborators to understand an unfamiliar condition. Together, these factors led to some interesting outcomes in proposal writing but overly ambitious and misaligned aims, ultimately resulting in an unfunded application. Considerable evidence suggests that team training does impact team effectiveness [44,45] and team interventions do affect team evolution and performance [48–50].

Although our understanding of the process of assembling a multi-institutional, translational research team is viewed through the experience of 40% of the participants in a case study format, the findings and suggested best practices form a solid basis for subsequent research on the phenomenon. Our findings emphasize the value of translational teams utilizing resources from Team Science that are available to them when forming a new team, either through educational modules online or at institutional Team Science Cores housed at CTSAs. Taking the time to align as a group, even on an abbreviated timeline, can yield great benefits in terms of team functionality and success. This is an enduring lesson from Team Science that held true in the case of this MCTT.

## Supporting information

Kotarba et al. supplementary material Kotarba et al. supplementary material

## Supplementary material

The supplementary material for this article can be found at https://doi.org/10.1017/cts.2023.640.
