# Supplementary material for: Exploring team dynamics during the development of a multi-institutional cross-disciplinary translational team: Implications for potential best practices
Source: J Clin Transl Sci. 2023 Oct 2;7(1):e220. doi: 10.1017/cts.2023.640 (PMC10643934; doi:10.1017/cts.2023.640)
Supplement: Kotarba et al. supplementary material [file S2059866123006404sup001.docx]

**Appendix A: Initial Interview Protocol**

1. How would you conceptualize an ideal biomedical research team? What values should be promoted in this research team? (Probe: Is this based upon your experience or your scholarly understanding? Does the MCTT fit your model?
2. How was the idea of the PASC MCTT presented to you and what were your thoughts and responses to this concept? (Probe: This is the wave of the future, or, this is our way of attracting funding for COVID-19 research?)
3. What were your initial expectations of how the MCTT would work out in terms of the way it would function?
4. How did you believe the context of the pandemic will influence the research study? (Probe: would a non-covid research project be treated differently? Is social urgency and need impacting the research?)
5. At the beginning of the project, what were your initial thoughts of how you would fit—personally and scientifically—in the team?
6. How did you expect your relationships and interactions with team members from the other institutions would transpire over time?

**Appendix B: Follow-up Interview Protocol**

1. How were you informed of the results of the first review?
2. What was your initial reaction to the review?
3. Did you discuss the first review with colleagues, etc.?
4. Did you feel any responsibility for the first review results?
5. What explanations were given you for the first review?
6. What do you feel were the “critical events” that resulted in no score?
7. What are the expected “critical events” determining scores on proposals?
8. What were the take-aways (personal or scientific) for you from that experience?
9. How would you approach your involvement with a second proposal?
10. How do your feelings now compare or contrast with those during the proposal assembly?
11. Have you maintained contact/friendships with teammates from the first proposal?
12. What have you learned about scientific “teams” or “teaming” from the first proposal?
13. What would you specifically like to see in the future work of the MCTT team? (Probes: relationships, work procedures, leadership formation or change, dissolution, best practices, etc.)
